# Signatures of a macroscopic switching transition for a dynamic microtubule

**DOI:** 10.1038/srep45747

**Published:** 2017-04-04

**Authors:** J. S. Aparna, Ranjith Padinhateeri, Dibyendu Das

**Affiliations:** 1 Centre for Research in Nanotechnology and Sciences, Indian Institute of Technology Bombay, Mumbai, India; 2 Department of Biosciences and Bioengineering, Indian Institute of Technology Bombay, Mumbai, India; 3 Department of Physics, Indian Institute of Technology Bombay, Mumbai, India

## Abstract

Characterising complex kinetics of non-equilibrium self-assembly of bio-filaments is of general interest. Dynamic instability in microtubules, consisting of successive catastrophes and rescues, is observed to occur as a result of the non-equilibrium conversion of GTP-tubulin to GDP-tubulin. We study this phenomenon using a model for microtubule kinetics with GTP/GDP state-dependent polymerisation, depolymerisation and hydrolysis of subunits. Our results reveal a sharp switch-like transition in the mean velocity of the filaments, from a growth phase to a shrinkage phase, with an associated co-existence of the two phases. This transition is reminiscent of the discontinuous phase transition across the liquid-gas boundary. We probe the extent of discontinuity in the transition quantitatively using characteristic signatures such as bimodality in velocity distribution, variance and Binder cumulant, and also hysteresis behaviour of the system. We further investigate ageing behaviour in catastrophes of the filament, and find that the multi-step nature of catastrophes is intensified in the vicinity of the switching transition. This assumes importance in the context of Microtubule Associated Proteins which have the potential of altering kinetic parameter values.

Microtubules are highly dynamic self-assembled structures in living cells having multiple functions including chromosome segregation, cellular transport and active maintenance of cell structure^1^,^2^,^3^. It has been experimentally observed that a growing microtubule filament stochastically switches to a state where it rapidly shrinks (exhibiting “catastrophe”) for a while, and reverts to a growing state (exhibiting “rescue”), under appropriate conditions. This dynamic spectacle, known in the biology literature as dynamic instability^4^, is a phenomenon far away from thermodynamic equilibrium as it is a consequence of the irreversible chemical processes of hydrolysis.

Earliest theoretical descriptions of dynamic instability involved assuming catastrophes and rescues as events with single timescales. Considering growth/shrinkage velocities and catastrophe/rescue frequencies as input parameters, these models studied various aspects of the system^5^,^6^. Later experiments revealed that catastrophe events are preceded by multiple kinetic events^7^,^8^,^9^, and hence necessitated a more microscopic approach to study the phenomenon. These microscopic models can be broadly categorised into two types: (i) models that account for details of structural and mechanical aspects of the protofilaments and the interactions between them^10^,^11^,^12^,^13^,^14^,^15^,^16^,^17^, and (ii) models that use a coarse-grained approach as far as multi-protofilament nature and elastic interactions are concerned but explicitly account for detailed kinetic events such as polymerisation, depolymerisation and hydrolysis of subunits (refs 18, 19, 20, 21, 22, 23, 24 and this work).

Despite a vast body of literature, no theoretical model can quantitatively explain all the features associated with microtubule catastrophes and rescues^25^. Moreover, there are unexplained variations in estimates (over two-orders) of depolymerisation rates of GTP-tubulins^26^, which may potentially arise from large fluctuations near critical concentrations. Very little has been understood about the non-equilibrium nature of the phenomenon of dynamic instability and how one should analyse the fluctuations in this system given its complex dynamics.

In our opinion, a physical understanding of the phenomenon of dynamic instability in microtubules needs to go beyond explaining the precise values of frequencies of catastrophes and rescues and their mechanism. One of the fundamental questions is whether there is co-existence of multiple “macroscopic” velocity phases in subpopulations of an ensemble of filaments, and what is the nature of transition between these macroscopic velocity states. By “macroscopic” velocities, we refer to the coarse-grained velocities (over long time intervals) of filaments, and not instantaneous ones. Although earlier studies by Mitchison *et al*.^4^,^27^,^28^ had suggested that an ensemble of microtubules may show a sharp switch-like transition between the mean-growth and mean-shrinkage phases, no theoretical or experimental work, to the best of our knowledge, has addressed this problem using the appropriate language of non-equilibrium phase transitions. Individual microtubules have been observed to switch between states of rescue and catastrophe. In contrast, here our concern is a different switching of macroscopic velocity state of an ensemble of filaments. Would a microscopic model inclusive of individual kinetic events (polymerisation, depolymerisation and hydrolysis) give rise to an abrupt switching transition in mean growth velocity^27^ with changing tubulin concentration? If such a transition can be captured using a theoretical model, it would be of interest to probe conditions for which the discontinuity and the associated co-existence of the two phases would emerge. These questions assume further significance since one would like to know under what conditions Microtubule Associated Proteins (MAPs) and chemical drug molecules would cause microtubules to switch between macroscopically stable and unstable states.

In this paper, we study a model which is better equipped to address many of these issues. We find that the language of phase transitions becomes necessary and useful in understanding the complex dynamics of the model-filament. Kinetic Monte-Carlo simulations^29^ reveal characteristics which approach that of an underlying discontinuous transition. We note that unlike one-dimensional (1-*d*) equilibrium systems^30^, nothing prohibits non-equilibrium 1-*d* systems to exhibit phase transitions^31^,^32^,^33^,^34^. Our systematic study of this question shows that while a true transition in the thermodynamic limit maybe absent in the biophysically relevant parameter regime *in vitro*, yet the quantitative extent of abruptness and discontinuities of the transition are large enough to warrant serious experimental attention. In particular, the large changes caused by small concentrations of drugs and MAPs on microtubule kinetics, may be seen in a new light of discontinuous transitions. We further observe that in the parameter regime that produces the abrupt transition, the model produces realistic values of length fluctuations and catastrophe frequencies with regard to experiments. Also, the multi-step mechanism of catastrophe, which manifests itself through time-dependent catastrophe frequencies^7^,^9^, is enhanced in this regime. Below we first define the model, study the underlying discontinuous transition and its implications, and proceed to present the results in comparison to experiments on age-dependent multi-step catastrophes.

## Methods

### The model

**Figure 1:**
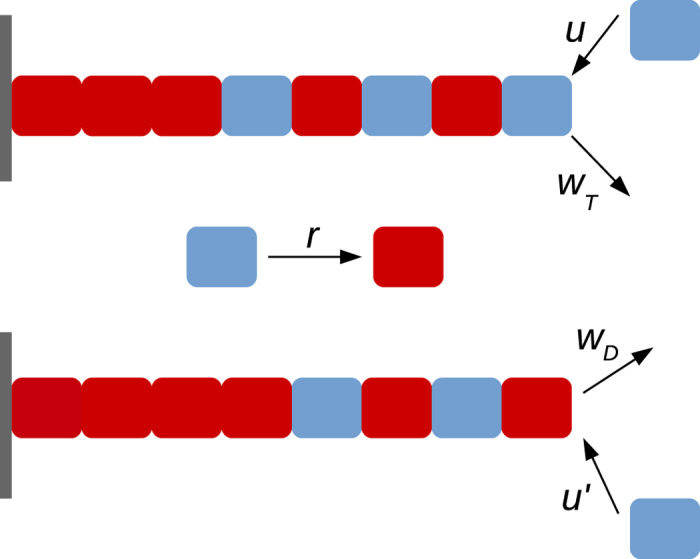
Schematic representation of the model. The polymerisation and depolymerisation rates are different when the tip is GTP-bound (grey, see top panel), and GDP bound (red, see the bottom panel). The hydrolysis which may happen anywhere on the filament, randomly, is shown separately in the middle panel.

Figure 1 depicts different microscopic kinetic events in our model of a single filament. The filament is immersed in a bath of GTP-associated tubulin subunits at a fixed concentration *C*. The GTP-subunits from the solution polymerise with a rate of *u* = *kC*, where *k* is the intrinsic rate of polymerisation when the filament tip is in GTP-bound state. *w*_*T*_ and *w*_*D*_ are the rates of depolymerisation of the GTP bound subunits and GDP bound subunits, respectively. Within the filament, the GTP molecule can hydrolyse into GDP and the mechanism of hydrolysis is assumed to be random–i.e., a GTP-subunit at any random position along the filament can hydrolyse with a rate *r*. As a result, the filament may consist of a ‘cap’ of GTP-subunits at the growing end of the filament with islands of GTP-subunits throughout the filament^35^. Since *w*_*T*_ ≪ *w*_*D*_, the GTP-cap at the end of the filament is considered to stabilise the polymer^26^,^36^. The loss of the cap exposes the GDP bound subunits at the end of the filament. Experimental evidences, in accordance with the structural distinction between the GTP-subunit and GDP-subunit, indicate that if the free end of the filament is GDP-rich, the end curves out like a ram’s horn^37^,^38^. This curving out behaviour and related physical features make a microtubule less favorable to polymerise and grow, when its tip is in a GDP-bound state. Consequently, in our model, polymerisation of GTP-subunits on a GDP-bound tip occurs at a rate *u*′ = *k*′*C*, which may be much lower than the rate *u* defined above.

Note that we are using a coarse-grained model here as it is nearly impossible to obtain statistical properties with sufficiently long time- and large ensemble-averages from detailed models (due to computational cost). A recent review by Howard and coworkers^25^ highlighted the importance of such coarse-grained models. The detailed models often involve many assumed interaction parameters which are not known experimentally. The coarse-grained models have fewer parameters, provided by experiments. Below we present the results by studying the model using kinetic Monte Carlo simulations.

The values of the kinetic parameters used for our quantitative study were obtained from earlier experimental estimates^2^,^4^,^26^,^39^. We use *k* = 3.2 *μM*^−1^*s*^−1^, *w*_*T*_ = 24 *s*^−1^, *w*_*D*_ = 290 *s*^−1^, *r* = 0.5 *s*^−1^. We also use a parameter *γ* (≈0.1 − 0.2 *μMs*^−1^) to denote the rate of change of tubulin concentration, in Results section under the heading ‘*Existence of hysteresis*’.

## Results

We use kinetic Monte-Carlo simulations^29^ to generate the length versus time traces of dynamic microtubule filaments. From these length versus time data obtained for various tubulin concentrations *C*, we calculate coarse-grained velocities for individual filaments, averaging instantaneous velocities over time intervals *T*. Such coarse-grained velocities serve as the sample set of order parameter values obtained from an ensemble of ~10^3^–10^5^ filaments. This sample set is used to study distribution and various cumulants of the velocities. In the subsection under the heading ‘*Signatures of transition studied using kinetic Monte-Carlo simulations*’, we investigate whether the distributions and the cumulants show signatures characteristic of a phase transition. In subsection titled ‘*Age-dependence and multi-step nature of catastrophes*’, the biophysical relevance of the model is discussed by studying the catastrophe frequencies and their age-dependence vis-a-vis the existing *in vitro* experimental data. In particular, the existence of a multi-step catastrophe process is probed.

### Signatures of transition studied using kinetic Monte-Carlo simulations

#### Mean velocity versus concentration

**Figure 2:**
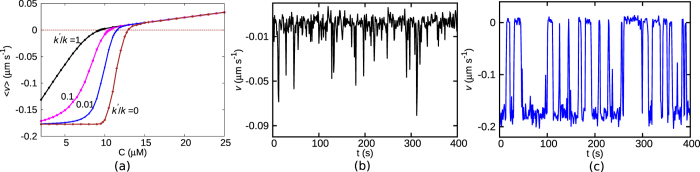
(**a**) Mean velocity 〈*v*〉 as a function of concentration *C* for *k*′/*k* = 0 (brown), 0.01 (blue), 0.1 (magenta) and 1 (black). The time series of the instantaneous velocity of the filament at (**b**) *C* = 9.4 *μM* for *k*′/*k* = 1 and (**c**) *C* = 9.7 *μM* for *k*′/*k* = 0.01.

In Fig. 2(a), we plot the mean growth velocity 〈*v*〉 (computed for *T* = 200 s) as a function of *C* for different *k*′/*k* values. With an increasingly lower likelihood of polymerisation on a GDP-bound tip (i.e *k*′/*k* → 0), the nature of the 〈*v*〉 curve changes-from a slow crossover (see *k*′/*k* = 1) to an increasingly abrupt switch-like behaviour (see *k*′/*k* ≤ 0.01). For the latter case, the velocity changes from a positive to a negative value within a relatively short range of concentrations. Near the respective transitions, the time series of the instantaneous filament velocity *v(t*) also shows a dramatic change. For *k*′/*k* = 1, the curve *v(t*) has only occasional and short-lived excursions to negative values (Fig. 2(b)). While for *k*′/*k* = 0.01, the curve for *v(t*) shows clear toggling between two states with positive and negative velocities (Fig. 2(c)). This is reminiscent of a discontinuous phase transition exhibiting co-existence of two macroscopic states. This is the first signature which supports the suggestion made by Mitchison and Kirschner^27^, which was not explored further in subsequent single MT filament experiments (see the first subsection under Discussion). Also note that, the well-known model by Dogterom *et al*.^5^ does not show such an abrupt switching in the mean velocity, as discussed under the title *‘Comments on earlier models in the context of the current work’* under Discussion.

#### Distribution of coarse-grained velocity and its cumulants

**Figure 3:**
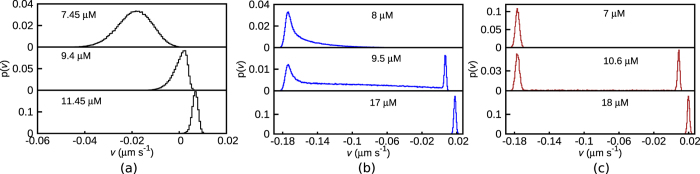
The probability distribution *p(v*) of coarse-grained velocities *v* (for *T* = 20 s) for (**a**) *k*′/*k* = 1, (**b**) *k*′/*k* = 0.01 and (**c**) *k*′/*k* = 0 showing the continuous versus discontinuous transition scenarios.

In Fig. 3, we plot the distribution *p(v*) of coarse-grained velocities *v* with *T* = 20 s. For *k*′/*k* = 1, as the concentration decreases, the peak of the distribution continuously shifts from positive to negative values of *v* (Fig. 3(a)). To the contrary, for *k*′/*k* = 0.01 (Fig. 3(b)), as *C* is lowered, over a small range of concentrations (≈10 ± 1 *μM*), a negative peak emerges and co-exists with the positive peak. For example, a bimodal distribution is shown for *C* = 9.5 *μM* for *k*′/*k* = 0.01 (Fig. 3(b)). As *C* is lowered further the positive peak disappears, and the distribution is dominated by negative velocities. Similar behaviour persists for the limit *k*′/*k* = 0 and the bimodality in velocity distribution at *C* = 10.6 *μM* is plotted in Fig. 3(c). Note that microscopic switches from growth to shrinkage are seen for all values of *k*′/*k* ∈ [0, 1]. Yet only for very small 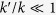 do we see a co-existence (i.e. bimodality) of two macroscopic velocity states. This distinction between microscopic toggling versus long-lived macroscopic switching is an important feature of our model, and may have implications in understanding the behavior of MAPs, drugs and motor proteins (further elaborated in the last two subsections of Discussion).

**Figure 4:**
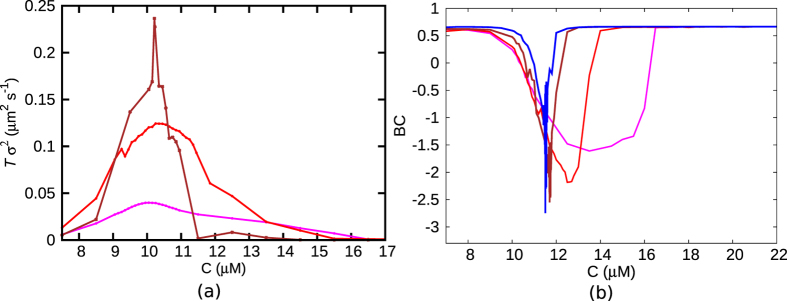
(**a**) *Tσ*^2^ and (**b**) Binder cumulant (BC) are plotted as a function of *C* for various values of *T* (magenta-*T* = 20 s, red-*T* = 50 s, brown-*T* = 200 s, blue-*T* = 500 s), for *k*′/*k* = 0.01. (**a**) Near the transition, *Tσ*^2^ shows a tendency of divergence, which is enhanced as *T* increases. (**b**) The sharp negative dip (blue) in BC indicates a discontinuous transition. As *T* increases, the dip in BC gets sharper.

The jump of the peak of the distribution (Fig. 3(b)), with changing *C*, would manifest in large fluctuations of the velocity ‘order parameter’ at the transition. These fluctuations would leave their mark on the observable cumulants of the distribution. Going beyond the first cumulant (〈*v*〉), we also compute the second cumulant/variance of velocity *σ*^2^ = 〈*v*^2^〉 − 〈*v*〉^2^. The scaled variance (*Tσ*^2^) shows a tendency of divergence, which gets sharper with increasing *T* (see Fig. 4(a))–note that *T* is the equivalent of system volume in spatially extended systems^40^.

A sharp switch in 〈*v*〉 and a sharp peak in *Tσ*^2^, as seen above, are reminiscent of a discontinuous phase transition. We now study the Binder cumulant (BC) defined as 1 − [〈*v*^4^〉/3〈*v*^2^〉^2^], a well-known quantity that is used to distinguish between discontinuous and continuous transitions^40^,^41^,^42^. BC is supposed to have a smooth crossover at a continuous transition. However at the point of emergence of a double peak at a discontinuous transition, BC is expected to have a sharp negative dip^40^,^41^. For very large or very small *C* values, the observed value of *BC* ≈ 2/3 (in Fig. 4(b)) may be explained by approximating *p(v*) as a delta function. But the interesting thing is that in the same range of *C* where 〈*v*〉 shows an abrupt switch, we see BC dipping sharply to negative values (in Fig. 4(b)) supporting yet again a discontinuous transition like scenario. Note that the extent of dip enhances with increasing *T* which is the expected standard finite system size behaviour^40^,^42^. We have checked that similar characteristics extend to *k*′/*k* = 0 limit–see Figure. S2 of the Supplementary Information for *Tσ*^2^ and BC for this limit.

#### Existence of hysteresis

**Figure 5:**
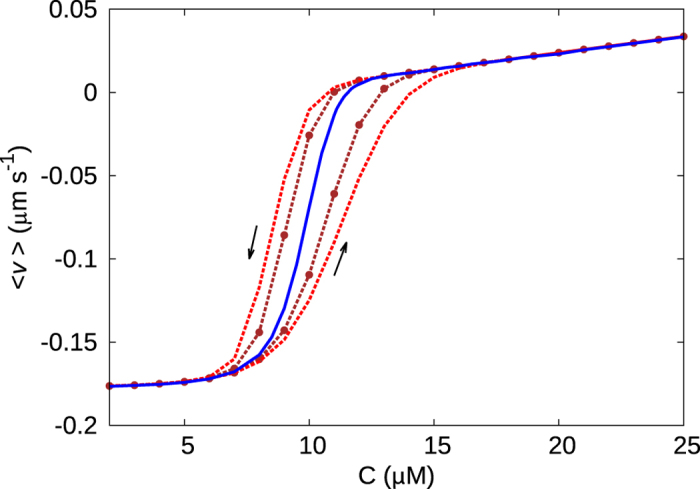
A hysteresis curve is observed for *k*′/*k* = 0.01 with ramping rate *γ* = 0.2 *μMs*^−1^ (red dashed lines) and 0.1 *μMs*^−1^ (brown dashed lines with circles). The upward and downward arrows indicate the paths corresponding to increase and decrease in *C*, respectively. The blue central curve is for *γ* → 0 (steady state).

The bimodality in the velocity order parameter (see Fig.3(b)) in the steady state points to the possibility of memory effects in the dynamical behaviour of a microtubule. Such a memory effect typically manifests in a *hysteresis* curve, for time-dependent changes of concentration. It is expected that a fast change in concentration would not allow the system to respond immediately to attain the probabilistically dominant value of *v*, because of the underlying bimodality and associated kinetic barriers. We track the velocity *v* on changing the concentration *C* of the system at a *finite rate γ*. In Fig. 5, we see that the curve of *v* for ramping up of *C* is distinct from the one corresponding to the ramping down of *C* (see the arrows). Furthermore larger the value of *γ*, i.e. lesser the time for the system to adjust to global parameter changes, the bigger is the hysteresis loop area. We would like to note that existence of hysteresis is often associated to discontinuous transitions^40^. Such memory effects may be interesting to study experimentally using a setup of temporally regulated flow of tubulin monomers.

### Age-dependence and multi-step nature of catastrophes

**Figure 6:**
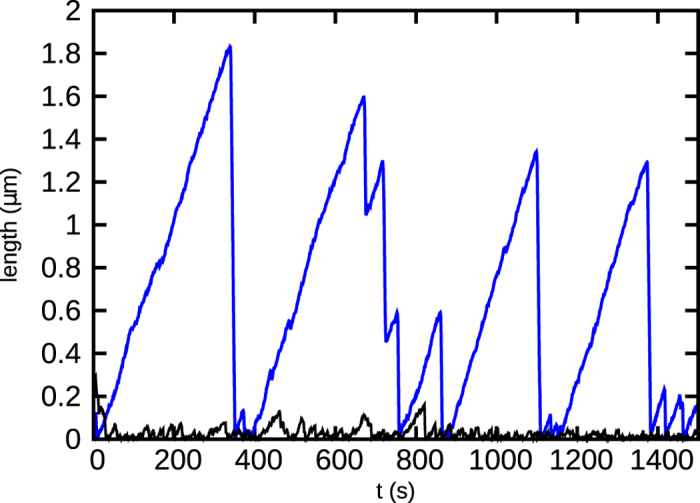
The length of a filament as a function of time for *k*′/*k* = 0.01 (blue) and *k*′/*k* = 1 (black). For *k*′/*k* = 0.01, the magnitudes of length and time compare well with known experimental data^9^,^43^,^47^. Concentration of free tubulin is 0.95C^*^, where C^*^ = 11.54 *μM* for *k*′/*k* = 0.01 and C^*^ = 9.4 *μM* for *k*′/*k* = 1.

So far we have discussed how the random hydrolysis model with variable polymerisation rate can generate signatures of a discontinuous transition from the mean growth to mean shrinkage phases of microtubules. However, it remains to be explained how well the above model compares with *in vitro* experiments. Firstly, by suitable choice of values of the polymerisation rate ratio *k*′/*k*, our model produces similar length versus time traces (i.e. kymographs) as seen in experimental studies of microtubules. Figure 6 shows two such comparative traces for a single microtubule filament, with *k*′/*k* = 0.01 (blue) and *k*′/*k* = 1 (black). Both these curves are plotted at concentrations just below their respective critical concentrations C^*^ (i.e., *C* = 0.95C^*^, where C^*^ = 11.54 *μM* (blue) and C^*^ = 9.4 *μM* (black)). While the black curve corresponding to *k*′/*k* = 1 does not produce experimentally valid time traces, the scales of the catastrophe events for *k*′/*k* = 0.01 (blue) are of similar order as observed in published *in vitro* experiments^9^,^43^. For example, the kymographs plotted in Gardner *et al*.^9^ show catastrophe events with typical catastrophe-times ranging from 200–500 s and lengths upto a few *μm* s – kymograph in Fig. 6 (blue) shows similar length- and time-scales of catastrophes.

It is important to note that (as discussed in details under Discussion) many earlier theoretical studies often used ad hoc criteria to identify catastrophes, or could not generate long time traces due to their computationally demanding methods. A key feature of our work is that we measure catastrophe times directly from the length versus time data, as done in experiments. We define a catastrophe event as a shrinkage larger than ≈500 nm in filament length.

**Figure 7:**
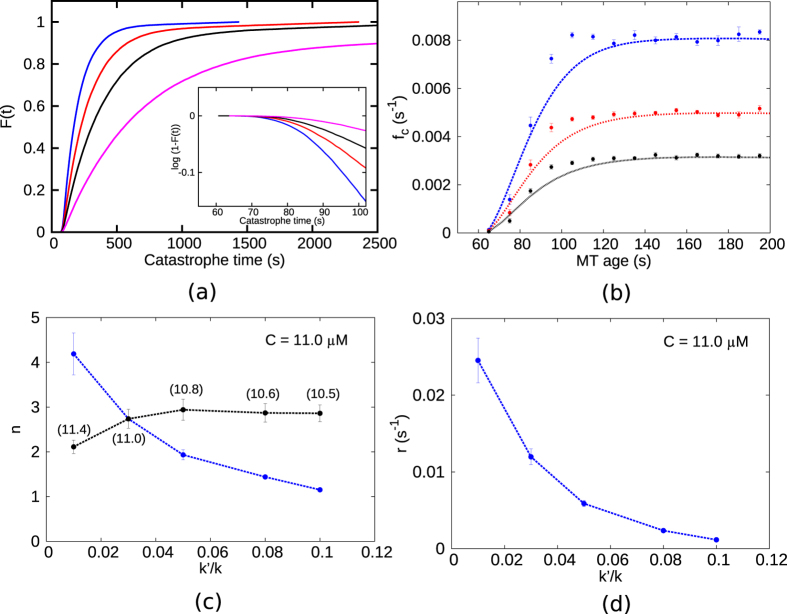
(**a**) Cumulative distribution of catastrophe times (*F(t*)) for values of *k*′/*k* = 0.01 (blue), 0.03 (red), 0.05 (black) and 0.08 (magenta). Inset (same color code) shows a nonlinear relation between *log*(1 − *F(t*)) and *t* indicating the multi-step nature of catastrophe. (**b**) Frequency of catastrophe (*f*_*c*_) as a function of microtubule age (same color code as in (**a**)). The absence of a single frequency for all time points is evident. Points shown are averages over a sliding window of 10 s. Error bars represent standard errors in *f*_*c*_ within the sliding window. (**c**) The minimum number of steps (*n*) that lead to an event of catastrophe at various values of *k*′/*k*. The blue curve is at *C* = 11 *μM*. The black curve corresponds to *n* measured at concentrations *C* = 0.99C^*^, where C^*^ is the critical concentration corresponding to each *k*′/*k*. The exact values of *C* corresponding to each *k*′/*k* are mentioned beside each point in the curve. (**d**) The rate *r* corresponding to each of the multi-steps is plotted as a function of *k*′/*k*. In (**c**,**d**), error bars correspond to 95% confidence intervals.

Drawing motivation from recent studies that investigate “age-dependence” of catastrophe frequencies^9^, we compute a cumulative distribution *F(t*) of catastrophe times *t. F(t*) is defined as the fraction of filaments having undergone catastrophe by time *t* from the beginning of the simulation. For different values of *k*′/*k, F(t*) is plotted in Fig. 7(a). For a single-step catastrophe process, it is expected that *F(t*) = 1 − *e*^−*st*^, where *s* is the rate of the step involved. To test whether the catastrophe is a single step event or not we plot *log*(1 − *F(t*)) in the inset of Fig. 7(a) and find that the curve is non-linear; this confirms that the catastrophe event in our model is not a single step but rather a multi-step event. The multi-step nature of the catastrophe we obtain from our model is consistent with the known experimental data and is in contrast with earlier theories that assume a single rate of switching from a growth to a shrinkage state.

Further, *F(t*) can be used to measure the catastrophe frequencies (*f*_*c*_) as defined in Gardner *et al*.^9^:


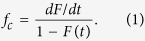


Frequencies calculated from Eq. 1 are plotted in Fig. 7(b) against microtubule ‘age’ (total time for which the filament has grown before undergoing catastrophe) at various values of *k*′/*k*. It can be seen from the figure that *f*_*c*_ shows an age-dependence similar to experiments^9^. The range of catastrophe frequencies we get (Fig. 7(b), at *C* = 11 *μM*) are also comparable to the data from experiments^9^ in the concentration range *C* = 10–12 *μM*. The *f*_*c*_ data seem to saturate at times smaller than in experiments, but this feature is similar to another theoretical model based on a multi-step mechanism of catastrophe^15^. Earlier models of microtubules^5^ that assume a single-step catastrophe would imply a *f*_*c*_ which is a constant, *for all times*.

In Gardner *et al*.^9^, the age-dependence of catastrophes was related to the underlying multi-step nature of catastrophes through an analysis of the probability distribution function *dF/dt*. By fitting the experimental data for the latter function to a Gamma distribution, they extracted quantitative measures related to the multi-step process. Starting with


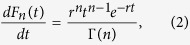


they found *n*, which is the minimum number of independent equal steps (each of rate *r*) required to trigger a catastrophe event. We have done a similar analysis using the simulation data plotted in Fig. 7(a), and fitted *dF/dt* to the gamma distribution in Eq. 2. In Fig. 7(c), the best fit obtained for the parameter *n* is plotted for different values of *k*′/*k* (see the blue curve corresponding to *C* = 11 *μM*). We see that for sufficiently small *k*′/*k* (for which we observed the switching transition in the previous subsection), the value of *n* ≥ 3, indicating that catastrophes become strongly multi-step events with decreasing *k*′/*k*. The fitted values of the parameter *r* in Eq. 2 for various values of *k*′/*k* are shown Fig. 7(d) for *C* = 11 *μM* – higher *r* for the smaller *k*′/*k* indicates enhanced instabilities induced by reduced polymerisation to GDP-tips.

Note that the critical concentration C^*^ varies with the *k*′/*k* value. So instead of analysing the behaviour for all *k*′/*k* at a fixed concentration, one may study *n* at different concentrations *C* close to C^*^ (namely *C* = 0.99C^*^) corresponding to each *k*′/*k*. We find (see the black curve in Fig. 7(c)) that such an analysis leads to *n* ≈ 3 over a broader range of *k*′/*k*. Values of *n* ≈ 3 were reported in experiments^9^ and also in a multi-protofilament based theoretical study^15^.

The underlying multi-step mechanisms of catastrophe may be affected in different ways in the presence of different destabilising MAPs, as each protein may alter microscopic kinetic events in unique ways. It was observed in the experiments by Gardner *et al*. that two different proteins Kip-3 and MCAK gave rise to two different trends of *f*_*c*_, *n* and *r* as their concentration was varied. The trends seen for various quantities in Fig. 7(b–d) as *k*′/*k* varies, may be thought to be related to effect of MAPs. This biophysical aspect is further discussed in the last subsection of Discussion.

## Discussion

### Experimental background motivating a search for a switching transition in a dynamically unstable microtubule

About 30 years ago, in a set of papers by Mitchison *et al*.^4^,^27^,^28^, dynamics of individual microtubules were studied using light microscopic immunocytochemistry and electron microscopy, going beyond the bulk assembly rate measurements using change of turbidity^44^. They reported simultaneous co-existence of sub-populations of growing and shrinking microtubules, even at concentrations slightly below the critical concentration^27^. Summarising the experimental facts, the authors plotted a *qualitative* curve (without any numerical scales) of bulk growth rate versus monomer concentration, indicating a switching transition (see Fig. 7 in Mitchison *et al*.^27^). Discussing the transition, the authors commented that “a small change in monomer concentration can produce a large change in polymer mass”. Following this study, we are not aware of any published *quantitative* experimental curve for mean microtubule velocity as a function of monomer concentration, obtained from individual filament dynamics. Hence, the curiosity remains to know experimentally whether the transition from bulk growth to bulk shrinking state is a *gradual* one or an abrupt one like a *switch*. Although the paper^27^ reported simultaneous co-existence of sub-populations of growing and shrinking filaments hinting towards a bimodal distribution of velocities over a narrow range of concentrations around the transition, they provided no quantitative data about the relative sizes of sub-populations as a function of tubulin concentration. In this paper, within the framework of a theoretical model, we demonstrate an abrupt switching transition and a co-existence of two macroscopic states, for dynamic microtubules.

Interestingly in Mitchison *et al*.^27^, it was envisaged that the system has a “true phase transition in the thermodynamic sense”. No subsequent work known to us, investigated the issue of a phase transition in any systematic way. Given contemporary general interest in the study of non-equilibrium phase transitions^31^,^32^,^33^,^34^, microtubule dynamic instability is an interesting non-equilibrium problem worth studying. In this paper we have explored the extent to which the observed switching transition within our model, carries the signatures of a true discontinuous transition.

In the context of contemporary experimental literature, it is of interest to study microtubule dynamics in the presence of different types of molecules that bind to the microtubule polymer (drug molecules, MAPs) and change its behaviour. They either stabilise or destabilise the microtubule by affecting its microscopic dynamics. While the *nature* of the macroscopic transition in microtubules remains an open question for experimental studies, it is also of great interest to examine if such a transition will be intensified or diminished in the presence of such microtubule-binding molecules.

### Comments on earlier models in the context of the current work

**Figure 8:**
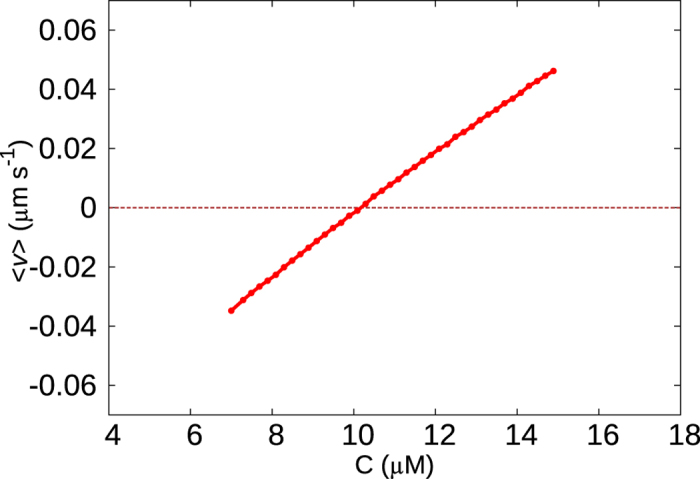
Mean growth velocity 〈*v*〉 as a function of tubulin concentration *C* measured for the phenomenological model of Dogterom *et al*.^5^ shows no abrupt switching behaviour. A similar time interval *T* = 200 s (as in Fig. 2) was used to coarse-grain length versus time data to obtain 〈*v*〉 values.

First, we would clarify that the switching behaviour we are talking of is not at a microscopic level but at a coarse-grained one. A popular phenomenological model due to Dogterom *et. al*.^5^ has a built in switch between two states (of positive and negative velocity), as *microscopic* rule of their model. It is interesting to note that if the velocities in that model are coarse-grained over a time interval *T* to extract the macroscopic growth/shrinkage behaviour, the model *does not show any abrupt switching transition*. There are four parameters in this model–the elongation rate, shortening rate, rate of switching from growth to shrinkage (catastrophe frequency) and rate of switching from shrinkage to growth (rescue frequency). These four parameters as a function of tubulin concentrations *C*, may be directly obtained from the experimental data of Walker *et al*.^43^. We simulated this model using kinetic Monte-Carlo for those experimental parameters. The mean growth velocities 〈*v*〉 (over timescale *T* = 200 s) at various *C*, are plotted in Fig. 8-as is clearly seen, 〈*v*〉 has no abrupt transition as it crosses from positive to negative value. Thus the model of Dogterom *et. al*.^5^ does not show the bulk switching behaviour as suggested by the experiment discussed above^27^. Note that the Dogterom model does not have the multi-step nature of catastrophe either. Interestingly, an earlier model by Hill *et al*.^45^ used a two state model like Dogterom *et al*., but with a different concentration dependence of shortening rate than measured in experiments^43^, and obtained a bend (or abrupt switching) in the mean velocity versus concentration plot; the study did not focus on the details of the transition and the resulting phase co-existence.

Even though there are many models that can successfully reproduce experimentally observed catastrophe frequencies and their multi-step nature, most of them have certain limitations. Hardly any of the models obtain events of catastrophes from long stretches of length versus time data. Moreover, some ad hoc criterion is often used to identify a catastrophe, like the occurrence of a particular microstate. For example, some models assume that once the tip of the filament becomes GDP-bound, catastrophe follows irrespective of the filament content (GTP/GDP spatial distribution) in the bulk of the filament^46^. Within such a model, a single timescale dominates the catastrophe frequencies contrary to the multi-step catastrophe observed in recent ageing experiments^7^,^9^. An improvement to this model, by incorporating multi-step catastrophe mechanism within a multi-protofilament model, was carried out by Jemseena *et al*.^15^. Here, while the catastrophe of a single protofilament (a ‘local catastrophe’) is assumed to be caused by the loss of GTP-tubulin at the filament tip (in a similar ad hoc fashion as ref. 46), the catastrophe of the entire microtubule results from a certain number of such individual protofilament catastrophes. Owing to the absence of depolymerisation of GDP-tubulin, they also do not present length versus time data. In another set of models^20^,^23^, a catastrophe event is considered to have occurred when the tip of the filament consists of “two” consecutive GDP-tubulins, which is again an ad hoc microstate. A molecular mechanical model by Zakharov *et al*.^16^ employs minimal assumptions regarding the definition of catastrophes and obtain catastrophe frequencies in agreement with experiments, but owing to computationally demanding Brownian dynamics techniques, they also do not produce long length traces.

In contrast, in our model, after defining the microscopic events–polymerisation, depolymerisation and hydrolysis–we do not make any more additional assumptions. We follow the length versus time data obtained as a result of these kinetic events (see Fig. 6) and see catastrophe emerging naturally with values which are similar to experiments. We note that the underlying microscopic events in our model are not ad hoc; apart from the well studied events described in ref. 21, we modify the polymerisation event depending on the state of the tip. Considering the well known experimental fact that protofilaments with GDP-bound subunits at the tip have a bent conformation^37^,^38^, it is reasonable to assume that such a conformation would make it less favourable to bind a new subunit. This assumption was used in certain other theoretical models^15^,^22^,^35^ too. Using the model, we have clearly demonstrated that tuning the differential polymerisation in GDP versus GTP tip-states (quantified by the rate ratio *k*′/*k*) can cause dramatic changes in the length traces of individual microtubules (see Fig. 6). In fact for sufficiently low values of *k*′/*k* we see length changes (~1–3 *μm*), and catastrophe times (~200–500 *s*) which are in the realistic range of numbers seen in experiments^9^,^43^,^47^. Figure 6 demonstrates that our assumption of 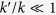 is necessary for this.

The 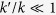 limit has been previously studied in a closely related model by Sumedha *et al*.^22^, where they investigate the dynamics of filament length in the presence of random hydrolysis mechanism. In the work^22^, it has been observed that the *k*′/*k* → 0 limit leads to a remnant dominated state of the filament, in which the presence of GTP-remnants within the filament regulates the length dynamics and leads to enhanced fluctuation. From our studies we observe that the signatures of discontinuous transition in growth velocity as well as the multi-step mechanism of catastrophes become prominent in the same limit. In the cap-dominated regime, the losing of the cap is the only rate-limiting step and hence catastrophe is likely to be single step (unless other complexities like multi-protofilament nature contribute^15^). To the contrary, in the remnant-dominated regime, multiple remnants can be present, and it is likely that they introduce many rate-limiting steps resulting in a multi-step mechanism of catastrophe as we observe for *k*′/*k*≪1.

A limitation of our model is that catastrophes do not prevail for a large range of concentrations as observed by *in vitro* experiments^9^,^43^. This limitation has been previously observed for other single filament models with random hydrolysis^25^. Hence, in the paper we have refrained from commenting on catastrophe frequencies far away from the critical concentration corresponding to each *k*′/*k*. It remains to be verified if major modifications of kinetic rates including differential mechanism of hydrolysis at the tip as compared to the interior of the filament, similar to what was done in ref. 25, would extend the concentration range in the model we used.

### Summary of results on the abrupt switching transition seen in our model

In this paper, we demonstrated the existence of an abrupt switching transition in the macroscopic dynamics of microtubule filaments within a theoretical model, consistent with suggestions of experiments^27^. Regulating the rate of polymerisation of a GTP-tubulin to a GDP filament tip (i.e *k*′/*k* → 0), the transition in mean velocity can be made sharper. Bimodality in velocity distribution indicating co-existence of two macroscopic positive and negative velocity states near the transition was demonstrated. Discontinuous transitions are accompanied by a localised divergence in variance, and a sharp negative dip in the Binder cumulant (related to the fourth cumulant)– we demonstrated these features for the microtubule system, similarly as it was done to characterise discontinuous transitions in other non-equilibrium systems^31^,^42^. Hysteresis effects were also shown, as expected in the vicinity of bistability.

Interestingly, in a recent work on the dynamics of a collection of microtubules attached to a kinetochore, bistability and hysteretic behaviour have been found^48^. While we study the kinetics as a function of tubulin concentration, Banigan *et al*.^48^ investigate co-existence of macroscopic velocity states as a function of an applied force. Based on the bistable collective motion of multiple microtubules attached to a kinetochore, the authors explain the center-of-mass and breathing oscillations of a pair of kinetochores.

A nice way to view our results is to compare it with the standard liquid-gas discontinuous transition in a fluid system. In Fig. S1 (Refer Supplementary Information), we have replotted the mean velocity 〈*v*〉 as a function of inverse concentration 1/*C* for various values of *k*′/*k* – for *k*′/*k*≪1, the curves show a striking similarity to liquid-gas isotherms below the critical point^49^. Thus 1/*C* is analogous to ‘pressure’, 〈*v*〉 corresponds to ‘specific volume’, and *k*′/*k* to ‘temperature’ of a fluid system. We have numerically checked that the discontinuous nature of mean velocity, and bimodality in velocity distribution disappear for *k*′/*k* larger than ≈0.4.

Our results show an abrupt switching transition in the microtubule system, but does this equate to a true discontinuous phase transition in the thermodynamic sense? The kinetic Monte-Carlo being an exact stochastic simulation, is reliable to capture all fluctuations. Yet with increasing coarse-graining intervals, the jumps, divergences and dips that we see in the first, second and fourth cumulants, respectively, are *not infinitely sharp*. This suggests that, possibly, in the “biophysically relevant” regime of parameters (chosen in our studies in conformity with *in vitro* experiments), the transition may not persist in the thermodynamic limit. However, this does not imply that the model would not give rise to a true transition for other values of parameters which we did not explore in our studies. A potential scenario is that a true thermodynamic transition exists in some other part of the parameter space, where for example the catastrophe time becomes unbounded providing a naturally divergent timescale. This may leave its imprint in the region of our interest. An extensive exploration of the parameter space would be a computationally demanding exercise which deserves a dedicated future research. In any case, regardless of the existence of a true phase transition, the quantitative magnitudes of discontinuities and abruptness are too large to escape suitably designed experiments, and are thus extremely relevant for future studies of the microtubule system.

### Biophysical significance of our results

In addition to intrinsic structural features, external molecules such as MAPs, motors or various drugs have been known to affect the dynamics of microtubules^9^,^38^,^50^,^51^,^52^,^53^. Microubule protofilaments were observed to curve out at the tip in the presence of XKCM1 proteins even when the tip tubulin contained slowly-hydrolysable GMPCPP^50^,^54^. Various anti-cancer drugs act by altering the dynamics of spindle microtubules^51^. Thus, external factors can modify the polymerisation/depolymerisation dynamics to varying extents and give rise to different growth kinetics and mean microtubule lengths. If the kinetic and dynamic properties of microtubules can be dramatically influenced by small changes in the external environment, this might have very significant biophysical consequences. If the 〈*v*〉 of the ensemble shows a bistability near the transition concentration, it implies that the system exists in a mean growth phase for a fraction of time and in a mean shrinking phase at other times. As evident from Fig. 2(a), for small values of *k*′/*k*, a slight change in *C* near the transition can change the macroscopic 〈*v*〉 of the ensemble by a significant amount (Δ〈*v*〉 ≈ 0.15 *μm/s*). This implies that if the ensemble of microtubules is in a state of co-existence, marked by enhanced dynamics, a small increase or decrease in concentration can push the system to a mean growing or mean shrinking phase, respectively. In addition, as seen in Fig. 2(a), a change in parameter *k*′/*k* can drastically shift the *C* versus 〈*v*〉 curve and also change the phase transition from being abrupt to gradual; this results in vastly different mean velocities for different values of *k*′/*k* at identical concentrations. Such scenario may have relevance in case of anti-mitotic cancer drugs which function by reducing the dynamics of spindle microtubules in order to impede cell division^51^.

External agents can also affect the multi-step nature of catastrophes by altering the mechanism which triggers catastrophe. This effect is probed in Gardner *et al*.^9^ using two destabilising proteins Kip-3 and MCAK. The presence of a stabilising/destabilising protein can affect the kinetics of a microtubule filament in various ways. A protein can bind to the microtubule and alter various kinetic rates^55^,^56^,^57^,^58^. Gardner *et al*. experimentally observed a variation in the mechanism of catastrophe in the presence of the two different proteins. This was inferred from the data for catastrophe frequencies *f*_*c*_, the step parameter *n*, and the rate parameter *r* as a function of the MAP concentrations. In the presence of Kip-3, *n* lies between 2–3 indicating a multi-step process. To the contrary, for MCAK, *n* decreases from ≈3 and approaches 1 as its concentration increases. Thus while for Kip3, an increase of catastrophe frequency is not associated with change in the mechanism of catastrophe, for MCAK the mechanism changes and approaches that of a single-step process with its rising concentration. In our model if a variation of *k*′/*k* is interpreted as the effect of variation of a certain MAP which actually regulates the rate of polymerisation to the GDP-bound tip of a filament, then our results indicate similar conclusions as above. For such a MAP whose rising concentration would effectively reduce the *k*′/*k* value, it would not only make the microtubule more unstable but also make the mechanism of catastrophe go from single to multi-step (Fig. 7(c) (blue curve)). This is a behaviour opposite to that of MCAK. Thus for such a MAP, which may be found by experimentalists in future, we have definitive predictions for the full age-dependent, multi-step, catastrophe behaviour.

In conclusion, in this paper we present the results of a macroscopic switching in microtubules, with associated signatures approaching the features of a discontinuous transition. Considering developments in the study of phase transitions in non-equilibrium systems in the past couple of decades, we think that our study of a macroscopic transition in microtubules using the language of non-equilibrium phase transitions is quite timely. In addition, the model suggests realistic history-dependence of catastrophes and their underlying multi-step nature, in the vicinity of the macroscopic transition. We hope that the suggested idea of regulating the transition using suitable MAPs would encourage interesting experiments in future.

## Additional Information

**How to cite this article**: Aparna, J. S. *et al*. Signatures of a macroscopic switching transition for a dynamic microtubule. *Sci. Rep.* **7**, 45747; doi: 10.1038/srep45747 (2017).

**Publisher's note:** Springer Nature remains neutral with regard to jurisdictional claims in published maps and institutional affiliations.

## Supplementary Material

Supplementary Information
