# Supplementary material for: Signatures of a macroscopic switching transition for a dynamic microtubule
Source: Sci Rep. 2017 Apr 4;7:45747. doi: 10.1038/srep45747 (PMC5379563; doi:10.1038/srep45747)
Supplement: Supplementary Information [file srep45747-s1.pdf]

Signatures of a macroscopic switching transition for a dynamic microtubule

Aparna J S, Ranjith Padinhateeri, Dibyendu Das, Indian Institute of Technology Bombay, India

Supplementary Information

S1. Phase diagrams

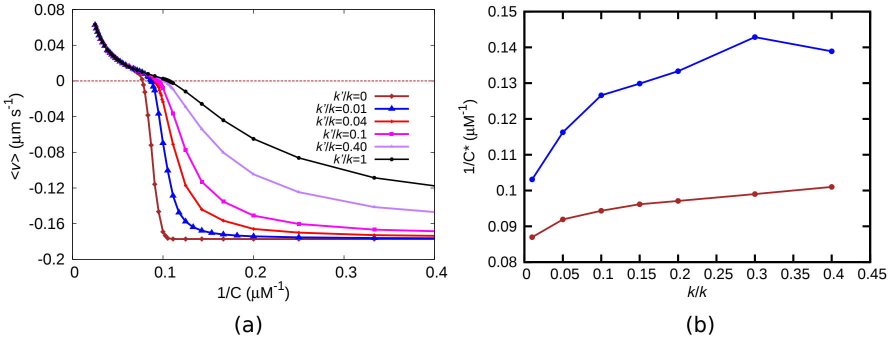

Figure S1

Although we have not done extensive study to draw a complete parallel between the discontinuous transition in microtubules as discussed in the manuscript, and the equilibrium liquid-gas phase transition, we indicate a few simple analogies here. Temperature, pressure and specific volume in a liquid-gas transition may be compared to the polymerisation ratio $k^{'}/k$, inverse concentration $1/C$ and mean velocity $\langle v\rangle$, respectively, in the microtubule system. The $1/C$ versus $\langle v\rangle$ curves (equivalent of the pressure-volume phase diagram) for various $k^{'}/k$ are plotted in Fig. S1(a). They are reminiscent of liquid-gas isotherms. The co-existence line of inverse concentration versus $k^{'}/k$ (equivalent of pressure-temperature co-existence line) is plotted in Fig. S1(b) - the lower brown curve is for critical concentrations C^{*} at which average velocity $\langle v\rangle$ vanishes, while the upper blue curve corresponds to the transition concentrations C_2^{*} at which $d\langle v\rangle/dC$ has maximum. Like liquid-gas system, where the first order transition pressure increases monotonically with transition temperature, here too we find similar monotonic increase of $1/C^{*}$ and $1/C_2^{*}$ as a function of $k^{'}/k$.

S2. Cumulants of velocity distribution for the limit $k^{'}/k = 0$

As mentioned in the manuscript, we plot the scaled variance ($T\sigma^2$) (Figure. S2(a)) and Binder cumulant (BC) (Figure. S2(b)) of velocity distribution as a function of GTP-tubulin concentration C, for $k^{'}/k = 0$. We note that the behaviour of these higher cumulants as 'system size' T becomes larger is qualitatively similar to that shown for $k^{'}/k = 0.01$ in Figure. 4 in the manuscript.

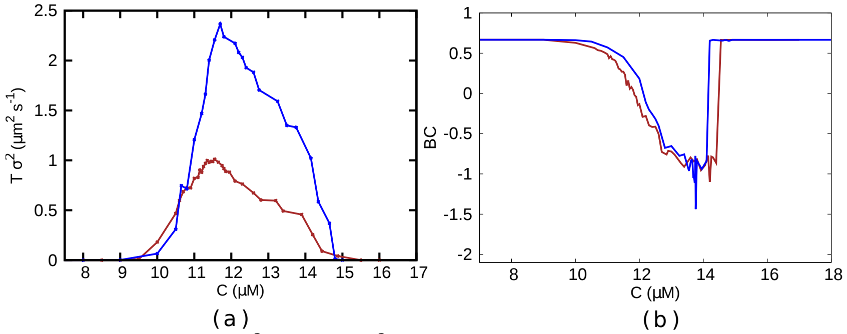

Figure S2. (a) Scaled variance $T\sigma^2$ (where σ^2 is the variance of the velocity distribution) is plotted against concentration C, for values of $T = 200s$ (brown) and $T = 500s$ (blue). It can be observed that as 'system size' T becomes large, $T\sigma^2$ tends to diverge. (b) Binder cumulant, BC (as defined in the manuscript) is plotted as a function of concentration C for values of $T = 200s$ (brown) and $T = 500s$ (blue). The sharp negative dip in BC for large T near the transition concentration is indicative of a discontinuous transition as discussed in the manuscript.
